# Assessing Market Food Diversity of Three Food Environments of Nairobi, Kenya, Using Spatial and Descriptive Analyses

**DOI:** 10.1007/s11524-025-00999-4

**Published:** 2025-09-08

**Authors:** Lucy Apiyo Adundo, Sofie Annys

**Affiliations:** 1 Justus-Liebig University Giessen, Giessen, Germany; 2 Antwerp University, Antwerp, Belgium; 3 Ghent University, Ghent, Belgium

**Keywords:** Food environment, Food group variation, Market food diversity (MFD), Spatial distribution of food vendors

## Abstract

The food environment (FE) is the interface where consumers interact to acquire and consume their food. Over the last two decades, the FE has changed due to lifestyle changes, globalization, economic disparities, and supermarketization. In addition, research on the food environment has focused mostly on high-income countries. Our study shows the novelty of assessing the food environment using the market food diversity (MFD) in Nairobi, a rapidly urbanizing city. We assessed the food environment of three income regions of Nairobi—using market food diversity derived from the Minimum Dietary Diversity of Women (MDD-W) tool. In August–December 2023, we used the Global Positioning System (GPS) and a list-based questionnaire to map 3548 food vendors. Descriptive statistics show that informal vendors (86%) dominated across the three income regions compared to formal vendors (14%). The high-income region was characterized by the presence of more formal vendors (24.2%) compared to the other income regions. There were statistically significant differences in food group variations among vendors. The study shows that unhealthy foods remain dominant across the three income regions. Market food diversity was highest in the high-income (3.11) region compared to the low- (2.71) and middle-income (2.35) regions. Our results underscore the need for policy implementation that supports local food environments that promote access to healthier and more nutritious diets, particularly in rapidly urbanizing cities of low- and middle-income countries (LMICs) such as Nairobi. Distinguishing income regions highlights the need for local policy officials to intervene to promote access to much healthier foods across the settlements.

**Supplementary Information:**

The online version contains supplementary material available at 10.1007/s11524-025-00999-4.

## Introduction

Food environment (FE) is defined as the consumer interface within the food system that encompasses affordability, availability, quality, convenience, promotion, and sustainability of foods and beverages, whether sourced from built environments, cultivated, or wild [23]. As an important component of the food system, the food environment significantly shapes consumer dietary choices [16] and influences nutrition transition [48, 52, 56, 59]. Urbanization is rising, especially in low- and middle-income countries (LMICs), with projections indicating that the urban population in these countries will exceed 4 billion by 2030 [27]. Sub-Saharan Africa (SSA) and Southern Asia are expected to experience the most rapid urbanization, with SSA projected to quadruple by 2025, reaching 1.3 billion compared to 306 million in 2010 [27]. These regions experience the highest rates of food insecurity, malnutrition, and inadequate access to affordable healthy diets [7, 15, 22].

The food environment is categorized into natural environments, including wild and cultivated food sources, and built environments, which consist of formal and informal market settings. Glanz et al. [28] propose a conceptual model that incorporates constructs related to individual healthy eating outcomes, giving higher priority to “community nutrition environment” and “consumer nutrition environment,” i.e., the built FE. The community nutrition environment refers to the type and location of food outlets such as supermarkets and tabletop vendors. The consumer food environment relates to what is available for consumers within each food vendor [28]. In addition, globalization, lifestyle changes, economic disparities, and the rapid growth of supermarkets have significantly altered the food environment over the past two decades, leading to significant shifts in urban diets [48, 52, 57, 59]. This has resulted in increased food and nutrition insecurity, including the triple burden of malnutrition [15]. Amid rising food and nutrition insecurity, food environments must be sustainable and healthy to enhance food and nutrition security [4, 33, 49, 51, 61].

The market food diversity (MFD) is a tool to measure food environments in LMICs. MFD is a metric derived from established food group benchmarks like the Minimum Dietary Diversity of Women (MDD-W) [2], p. 12). The MDD-W is a 10-point scale indicator used to assess individual diet quality and is recognized as a validated proxy for micronutrient adequacy [26, 36]. The MFD has been utilized in Tanzania to assess the relationship between FE and the dietary diversity of women, highlighting the availability of foods and food groups within markets [3]. The spatial distribution of food vendors is significantly influenced by the urban infrastructure, particularly busy roads, which as a result, which shapes vendor locations [29]. The linkage between vendors and food group variations is crucial, as different food vendors offer distinct food items that influence individual food choices and health outcomes [11, 43]. MFD of food environments is particularly important for policymakers as it influences consumer behavior and diets, impacting health outcomes [25, 44].

Despite evidence indicating that FE shapes diets, nutrition and health outcomes, much of that evidence originates from high-income countries [13, 35]. Studies in LMICs on FE and drivers of food choice (food access, availability, and affordability) have primarily focused on low-income areas, not giving much attention to middle- and high-income areas [1, 8, 10, 14, 19, 21, 46, 65]. Studies have shown differences in diversity of food outlets within low-income regions. Chege et al. [14] found that increased diversity of food outlets correlates with greater food availability. Supermarkets also offered a wider variety of foods such as fruits and vegetables, processed foods and dairy, while informal vendors mostly sold staples. Another study by Berger and van Helvoirt [6] examines the impact that retail modernization has on food security in Nairobi, in the context of increasing urbanization and evolving urban food systems. Additionally, a study investigates how food vendors shape the obesogenic food environment of an informal settlement (Kibera) in Nairobi [11]. This study combines descriptive and spatial analysis to examine how vendors influence the diets and health outcomes. Focusing on socioeconomic levels in LMIC contexts is crucial when evaluating the FE, as these disparities are likely to significantly influence consumer dietary choices and consequently related health outcomes [34, 38]. A comparison of MFD across the high-, middle-, and low-income regions is essential for creating a sustainable food environment that provides healthy and nutritious diets to all population segments.

The overall objective of the study is to assess the market food diversity across the three income regions of Nairobi. Our cross-sectional study represents state-of-the-art evidence, as to date, we are unaware of any research comparing high-, middle-, and low-income regions within an LMIC city. These empirical results uniquely contribute to the literature and ongoing debates on improving urban food environments. Like other LMI cities, Nairobi is facing a nutrition transition and dietary changes (from traditional to high-caloric) due to demographic change, rapid urbanization, advancements in technology, economic development, and supermarket expansion [32, 46]. Our findings have significant urban policy implications, particularly in Sub-Saharan Africa, as we shed light on urbanization and nutrition transition in urban areas. To research our study overall objective, the specific research questions include the following: (1) How are different vendor types (formal and informal) distributed across the different income regions?; (2) What is the linkage between vendor type and the variation in food groups?; (3) How does the MFD vary across different income regions?; and (4) What factors influence Market Food Diversity?

## Data and Methods

### Study Area and Sampling

Using a multi-method approach that provides a comprehensive understanding of the food environment [47], an extensive field survey was conducted in Nairobi, mapping the geographical distribution of food vendors and an inventory of the food items available at these locations was taken. The sampling strategy was based on the income regions. From the prescribed administrative boundaries by the Government, the following sub-counties were purposively selected: high-income (Kilimani), middle-income (Kasarani), and low-income (Makadara) [31, 40]. Within Kilimani, Kileleshwa and Kileleshwa wards were randomly selected. In Kasarani, Clay city, Kasarani and Mwiki wards were selected, while Viwandani ward was selected in Makadara. Vendor mapping aimed to exhaustively cover vendor locations in the study regions.

Local enumerators were trained to conduct data collection using the Open Data Kit (ODK). A list-based questionnaire of food groups was used for vendor audits/inventories assessing food and beverage availability. The food group list was adapted to include locally consumed food items. Food items recorded from each vendor were categorized into assigned food groups derived from the 10-scale dietary diversity food group of MDD-W [36].

Data were collected from August until December 2023. A total of 3458 food vendors were mapped: 508 in the high-income region, 1757 in the middle-income region, and 1193 in the low-income region. Data on the built food environment was collected using GPS vendor locations and vendor type characteristics.

### Overview of the Food Environment and Typology

We classified the vendors into two sub-types: formal and informal food environments [23]. Formal vendors operate on a license from the city-county authority, while informal vendors, such as kiosks, lack formal licenses but must pay a few for space usage. The following vendor types were considered: hypermarkets-/supermarkets, fast food outlets, modern restaurants, bakeries, farms, wet markets, ambulant/mobile vendors, poultry seller/butcher, cereal shops, and wholesalers following classifications from different authors and field observations [14, 23, 24, 62, 66] (Table 1).

**Table 1:** Food environment typology. Categorization of vendor types and access points within formal and informal food environments of Nairobi, Kenya

Built food environment		
Food environment type	Food environment sub-type	Description of food environment sub-typology
Formal food environments	Hypermarkets/supermarkets	Fixed structures. Large varieties of foreign and local brands; fresh, processed, and ultra-processed foods; non-food products. Also offer packaged foods such as cereals, legumes, milk, and milk products. Owned independently; self-service; payment through cashiers; no credit possibility
	Modern restaurant/fast food outlet/bakery	Casual dining, fast foods, including cafes where meals are prepared and sold for take-out, sit-down delivery or delivery
	Butcher	Fixed structure; licensed vendors that only sell meat and meat products, sometimes also fish
	Wholesalers	Sells dry food items to other small vendors; may also sell food items directly to customers
	Direct sale from farm	Government known; operates mostly daily and sells fresh dark green leafy vegetables directly from the farm
Informal food environments	Ambulant vendors/street hawkers/mobile vendors	No permanent infrastructure in the location; vendors travel (e.g., by motorbike, food truck, cart) from one point to the other selling food. Only present at specific times of the day, week, or month
	Cooked food street vendor/roadside catering	Fixed locations; operate long busy streets/roads; offer cooked food to customers; meals are prepared for take-out, sit-down delivery, or delivery
	Cereal shops	Small fixed structures that sell cereals and legumes in the form of grains and flour. Are individually- or family-owned; can offer credit to known customers
	Home vendors	Sell fresh foods within the homestead or home
	Kiosk/D*uka*	Small boutiques/small shops that sell food and non-food items; over the counter-service. Sell mostly processed and ultra-processed foods; credit possibility
	Mom-and-pop	Have fixed locations; offer a variety of foods and particular brands; small packaging; Refrigerated foods with small packaging; processed foods; Individual or family owned
	Poultry seller	Small mobile and temporary structures along the road; sell live chickens; may slaughter chicken upon request
	Tabletop vendors/stalls/*Mama mboga*	Small mobile and temporary stands along the road; sell fresh fruits and vegetables, roots and tubers. On request, can shred vegetables for customers; individually owned, mostly by women and youth; credit possibility to known customers
	Wet market*/open-air markets	Operation within fixed hours; clustered at specific points; operate daily or weekly in open-air settings; number of retailers differs according to operation hours or market days. Sell primarily fresh foods, dry cereals and legumes

*Source*: Adapted from Chege et al. [14], Downs et al. [23], Turner et al. [62], and Wanyama et al. [66] and updated based on field observations

^*^A wet market is a “fresh food market” that sells fresh fruits and vegetables, meat products, fish, and other consumption-oriented perishable goods that are not in a supermarket setting

### Computing the Market Food Diversity (MFD)

Following Ahmed et al. [2] and Ambikapathi et al. [3], we constructed MFD from MDD-W indicator to assess food (group) availability. MDD-W reflects diet quality and micronutrient adequacy [26]. The MFD is a count for measuring the market diversity (using food groups) in each income region. Initially, food items in the questionnaire were subdivided into seventeen food groups [26, 60], see Appendix (Table S1). To provide a comparable analysis, we constructed the MFD index by aggregating the 17 food group subdivisions into the 10 MDD-W food groups. While MFD serves as a metric for measuring the food environment, we acknowledge that certain metrics, such as food prices, were not captured to distinguish food affordability across regions.

From the MFD indicator, a four-scale MFD categorization was developed following the FAO [26] classification to distinguish different levels of market food diversities within the three food environments. An income region is considered more diverse when vendors achieve at least MFD ≥ 5 out of the 10 food groups.

### Estimating Market Food Diversity of Income Regions

Income regions—high-, middle, and low—play a crucial role in influencing MFD in urban food environments [68]. Each income region has distinct characteristics shaping vendor food availability. The relationship between MFD and income regions was tested using a partial Poisson regression model. Shelf food is significant within the food environment, as studies show that availability of certain foods, e.g., energy-dense foods, positively correlates with nutrition and health outcomes [55]. Vendor ownership and shelf food space were included as control variables in the partial Poisson model.

Ownership can significantly influence MFD as food vendors offer a range of food items based on consumer demand and expertise. Different vendor types may specialize in specific foods, affecting MFD depending on vendor type. For example, the variety of foods offered by a single-owned vendor may differ from that of a joint-owned enterprise [39].

Shelf food space was defined as the amount of space vendors allocated to food products [63]. For this study, space was classified into five groups: 100% food space, 75–99%, 50–75%, 25–50%, and below 25%. Food product allocation by a vendor determines the variety of food items available within a particular food group, influencing MFD within food environments.

Given our count outcome variable (MFD) and the categorical and binary nature of explanatory variables (income regions, vendor ownership, shelf food space), we used a Poisson model to assess this relationship. This was also informed by the right-skewed distribution observed in the MFD bar graph, making the Poisson model the most appropriate choice.

The model assumption is:

$$MFD \sim Poisson (\mu )$$

where *μ* is the mean, our econometric model is represented generically by the following equation:

1 $$log \left(\mu \right)= {\beta }_{0}+{\beta }_{1}{IA}_{1}+ {\beta }_{2}{VO}_{2}+{\beta }_{3}{FS}_{3}$$

where market food diversity is a count outcome variable; β_0_ is the intercept; β_1_, β_2_, and β_3_ are coefficients to be estimated; IA is the income area; VO is the vendor ownership and FS is the shelf food space.

To check for robustness of the model, we ran a generalized linear model (GLM) to compare its Akaike’s information criterion (AIC) with that of the Poisson model. AIC measures goodness of fit, considering the model’s ability to fit a dataset [9, 42]. The Poisson model had a lower AIC (11,876.35) compared to the GLM (AIC: 12560.42), indicating it was the most appropriate model for our dataset ().

### Statistical Analyses

A geospatial analysis of the recorded data was conducted using a Geographic Information System (GIS) to show the distribution of food vendors. ArcMap version 10.8.1. was used to map vendor locations and understand the geographical distribution of different MFD categories. Descriptive statistics (mean and Pearson’s chi-square test) were employed to describe food vendor characteristics (food group variations within each vendor type) and to test for differences within the characteristics. The Pearson chi-square was used to test the differences in MFD means in the three income areas. Descriptive statistical analyses were performed using the STATA 15.0 (StataCorp LP, USA) and R version 4.3.1 software.

## Results

### Spatial Distribution of Vendor Types

**Fig. 1:**
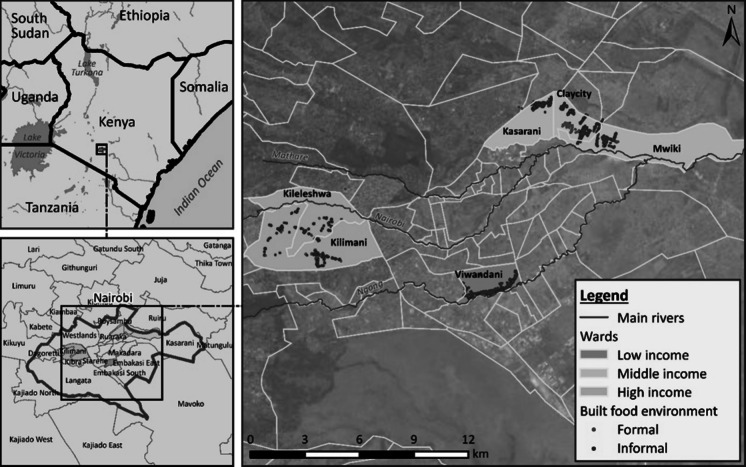
A map of built food environments showing formal (blue dots) and informal (purple dots) food vendors in Kilimani/Kileleshwa (high-income), Kasarani, Clay City and Mwiki (middle-income), and Viwandani (low-income) in Nairobi, Kenya (*n* = 3458)

We conducted a food environment study and collected data on 3458 vendor types. An illustration of the formal and informal food environment typologies shows that 14% of the mapped vendors were formal compared to 86% informal vendors. Informal food vendors dominate across all regions, with 95.2% of vendors in low-income areas being informal, compared to 82.7% in middle-income and 75.8% in high-income regions (Fig. 1).

Overall, the most common vendor types across all income regions were cooked food street food vendors or roadside caterers (25%), tabletop vendors/mama mboga^1^ (25%), kiosks (16.7%), and ambulant vendors or street hawkers (16%) (Table 2).

**Table 2:** Vendor distribution across income regions (percentages based on income region)

Vendor type	Overall, *N* = 3458^1^	Low-income, *N* = 1193^1^	Middle-income, *N* = 1757^1^	High-income, N = 508^1^
Ambulant vendor/street hawkers/mobile vendors	552 (16.0%)	116 (9.7%)	293 (17.0%)	143 (28.0%)
Butcher/poultry	188 (5.4%)	41 (3.4%)	133 (7.6%)	14 (2.8%)
Cooked food street vendor/Vibandanski/roadside catering	855 (25.0%)	378 (32.0%)	372 (21.0%)	105 (21.0%)
Farm/home vendor	79 (2.3%)	5 (0.4%)	74 (4.2%)	0 (0%)
Kiosk/duka	579 (17%)	319 (27.0%)	203 (12.0%)	57 (11.0%)
Mom-and-pop/cereal shop	106 (3.1%)	16 (1.3%)	79 (4.5%)	11 (2.2%)
Modern restaurant/fast food/bakery	169 (4.9%)	4 (0.3%)	78 (4.4%)	87 (17.0%)
Hypermarkets/supermarkets	36 (1.0%)	1 (< 0.1%)	17 (1.0%)	18 (3.5%)
Tabletop/mama mboga/stalls/wet market	861 (25.0%)	299 (25.0%)	493 (28%)	69 (14.0%)
Wholesalers	33 (1.0%)	14 (1.2%)	15 (0.9%)	4 (0.8%)

**Fig. 2:**
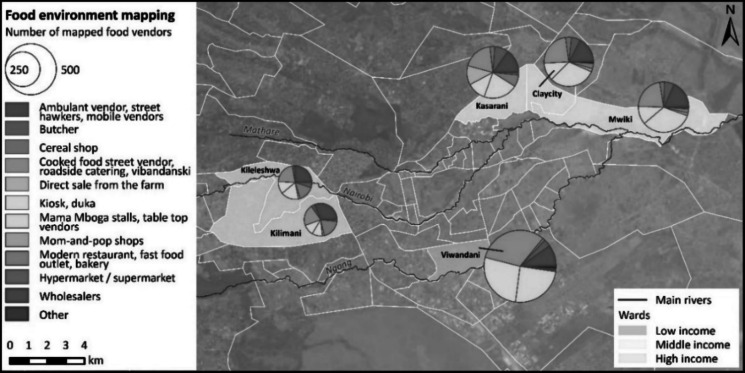
Typology map and distribution of vendors in Nairobi city. The size of the pie-chart indicates the aggregate number of vendors mapped in each ward across the three income regions

Relatively, most hypermarkets/supermarkets (3.5%) and modern or fast-food restaurants (17.1%) were found in the high-income region. In contrast, the low-income region was characterized by a greater presence of informal vendors, with cooked food street food vendors making up 31.7%, kiosks accounting for 26.7%, while tabletop vendors/mama mboga constituted 24.6% (also see Fig. 2). Ambulant vendors or street hawkers were less common, making up 9.7% of the vendors in the low-income. The remaining vendors in the low- and middle-income regions fell into other categories (farm, home vendor) (Fig. 2).

Results further show that the location of food vendors relative to road infrastructure varies significantly by income level. In the low-income region, most vendors are clustered along roadsides, whereas in middle- and high-income regions, vendors are more dispersed across various locations Fig. 1.

### Differences in Food Group Variations by Vendor Types Across Income Regions

To understand how each vendor type contributes to the food diversity within the urban food environment, the food groups provided by each identified vendor are presented in Table 3. Pearson’s chi-square test shows statistically significant differences in the variations of food groups among vendor types. Results show that the food group “Grains, roots and tubers,” a source of carbohydrates, was observed among 65% of all food vendors with the highest representation in hypermarkets/supermarkets (100%), mom-and-pop/cereal shops (98%) modern restaurants/fast foods (93%), and cooked food street vendors (86%). Legumes and pulses were observed among mom-and-pop/cereal shops (74%), modern restaurants (35%), and cooked food street vendors (30%). Most hypermarkets/supermarkets (92%) sold nuts and seeds and dairy (89%), though a significant stocking of dairy products (78%) was observed in kiosks. As expected, meat, poultry, and fish—important protein sources—are primarily stocked by butchers/poultry sellers, modern/fast food restaurants, and hypermarkets/supermarkets. Eggs (a protein source) were mostly available in hypermarkets/supermarkets (92%) and kiosks/duka (69%). Only 24% of all vendor types stocked dark green leafy vegetables, with farm/home vendors, tabletop vendors/mama mboga/wet markets, and hypermarkets/supermarkets being the most relevant suppliers of this food group. Additionally, vitamin A-rich fruits and vegetables were predominantly observed among tabletop vendors/mama mboga/wet markets (53%) and hypermarkets/supermarkets (42%). Among 30% of vendors who sold other vegetables, tabletop vendors/mama mboga/wet markets had the highest representation (75%), followed by hypermarkets/supermarkets (39%). Other fruits were sold by 25% of vendors, with the highest representation from tabletop vendors/mama mboga/wet markets (65%), and hypermarkets/supermarkets (42%).

**Table 3:** Food group variations by vendor types across income regions based on a sample size of 3286 vendor observations

Food groups	Overall, *N* = 3,286^1^	Ambulant vendor/street hawkers/mobile vendors, *N* = 497^1^	Butcher/poultry, *N* = 188^1^	Cooked food street vendor/roadside/Vibandanski, *N* = 837^1^	Farm/home vendor, *N* = 79^1^	Kiosk/duka, *N* = 523^1^	Mom&Pop/cereal shop, *N* = 103^1^	Modern restaurant/fast food outlet/bakery, *N* = 155^1^	Hypermarkets/supermarkets, *N* = 36^1^	TableTop/wet market, *N* = 841^1^	Wholesalers, *N* = 27^1^	*p*-value^2^
Grain, Roots and Tubers	2,134 (65.0%)	260 (52.0%)	25 (13.0%)	716 (86.0%)	15 (19.0%)	436 (83.0%)	101 (98.0%)	144 (93.0%)	36 (100%)	385 (46.0%)	16 (59.0%)	< 0.001
Legumes and pulses	570 (17.0%)	13 (2.6%)	2 (1.1%)	249 (30.0%)	1 (1.3%)	58 (11.0%)	76 (74.0%)	54 (35.0%)	19 (53.0%)	92 (11.0%)	6 (22.0%)	< 0.001
Nuts and seeds	392 (12.0%)	40 (8.0%)	0 (0%)	22 (2.6%)	1 (1.3%)	192 (37.0%)	52 (50.0%)	21 (14.0%)	32 (89.0%)	25 (3.0%)	7 (26.0%)	< 0.001
Dairy and dairy products	615 (19%)	17 (3.4%)	0 (0%)	41 (4.9%)	1 (1.3%)	406 (78.0%)	34 (33.0%)	52 (34.0%)	33 (92.0%)	17 (2.0%)	14 (52.0%)	< 0.001
Meat, Poultry and Fish	758 (23.0%)	47 (9.5%)	184 (98.0%)	318 (38.0%)	0 (0%)	8 (1.5%)	9 (8.7%)	126 (81.0%)	16 (44.0%)	50 (5.9%)	0 (0%)	< 0.001
Eggs	840 (26.0%)	74 (15.0%)	9 (4.8%)	150 (18.0%)	2 (2.5%)	363 (69.0%)	39 (38.0%)	75 (48.0%)	33 (92.0%)	86 (10.0%)	9 (33.0%)	< 0.001
Dark green leafy veg	778 (24.0%)	26 (5.2%)	4 (2.1%)	155 (19.0%)	66 (84.0%)	45 (8.6%)	10 (9.7%)	50 (32.0%)	14 (39.0%)	408 (49.0%)	0 (0%)	< 0.001
Vitamin A rich fruits and veg	571 (17.0%)	29 (5.8%)	0 (0%)	21 (2.5%)	0 (0%)	42 (8.0%)	8 (7.8%)	11 (7.1%)	15 (42.0%)	445 (53.0%)	0 (0%)	< 0.001
Other vegetables	991 (30.0%)	53 (11.0%)	1 (0.5%)	137 (16.0%)	4 (5.1%)	87 (17.0%)	9 (8.7%)	53 (34.0%)	14 (39.0%)	631 (75.0%)	2 (7.4%)	< 0.001
Other fruits	831 (25.0%)	110 (22.0%)	0 (0%)	51 (6.1%)	3 (3.8%)	69 (13.0%)	8 (7.8%)	24 (15.0%)	15 (42.0%)	549 (65.0%)	2 (7.4%)	< 0.001

^1^*n* (%) – The number of parentheses represents percentages of food vendors that offer each food group within their respective categories. 3286 is based on the MFD 10 food groups, excluding other additional food groups

^2^Pearson’s chi-squared test

### Food Vendors’ Food Group Variations Using MFD

The variations in food groups among the food vendors are further evaluated across the three income regions. Results in Table 4 show variations in the provision of MFD food groups within a vendor type across the income regions. This means that an individual vendor characteristic cannot be generalized within an urban FE. Ambulant food vendors are major providers of grains, roots, and tubers in the high-income region but their contribution in low- and middle-income regions is small. Additionally, farm/home vendors provide more grains. Roots and tubers are found in low- and middle-income regions, but their contribution to high-income is relatively small. The contribution of supermarkets to the supply of grains is relevant across high- and middle-income regions, but low in the low-income region as they are not very prevalent. Wholesalers provided more grains, roots, and tubers in the low-income region compared to the other two income regions.

**Table 4:** Food group variations among vendor types across income regions of Nairobi

Food groups	Vendor type/income regions											
	Ambulant vendor/street hawkers/mobile vendors, *N* = 265^*1*^			Butcher, *N* = 133^*1*^			Cooked food street vendor/roadside catering/Vibandanski, *N* = 360^*1*^			Farm/home vendor, *N* = 74^*1*^		
	Low-income	Middle-income	High-income	Low-income	Middle-income	High-income	Low-income	Middle-income	High-income	Low-income	Middle-income	High-income
Grain, roots and tubers	41 (41.0%)	125 (47.0%)	94 (71.0%)	7 (17.0%)	18 (14.0%)	0 (0%)	317 (85.0%)	302 (84.0%)	97 (93.0%)	4 (80.0%)	11 (15.0%)	No Obs
Legumes and pulses	2 (2.0%)	10 (3.8%)	1 (0.8%)	1 (2.4%)	1 (0.8%)	0 (0%)	133 (36.0%)	88 (24.0%)	28 (27.0%)	1 (20.0%)	0 (0%)	No Obs
Nuts and seeds	7 (7.0%)	13 (4.9%)	20 (15.0%)	0 (0%)	0 (0%)	0 (0%)	12 (3.2%)	7 (1.9%)	3 (2.9%)	1 (20.0%)	0 (0%)	No Obs
Dairy and dairy products	4 (4.0%)	10 (3.8%)	3 (2.3%)	0 (0%)	0 (0%)	0 (0%)	26 (7.0%)	12 (3.3%)	3 (2.9%)	1 (20.0%)	0 (0%)	No Obs
Meat, poultry and fish	2 (2.0%)	27 (10.0%)	18 (14.0%)	40 (98.0%)	130 (98.0%)	14 (100.0%)	138 (37.0%)	120 (33.0%)	60 (58.0%)	0 (0%)	0 (0%)	No Obs
Eggs	7 (7.0%)	45 (17.0%)	22 (17.0%)	1 (2.4%)	5 (3.8%)	3 (21.0%)	47 (13.0%)	54 (15.0%)	49 (47.0%)	2 (40.0%)	0 (0%)	No Obs
Dark green leafy veg	3 (3.0%)	21 (7.9%)	2 (1.5%)	2 (4.9%)	2 (1.5%)	0 (0%)	93 (25.0%)	42 (12.0%)	20 (19.0%)	1 (20.0%)	65 (88.0%)	No Obs
Vitamin A rich fruits and veg	8 (8.0%)	17 (6.4%)	4 (3.0%)	0 (0%)	0 (0%)	0 (0%)	11 (2.9%)	8 (2.2%)	2 (1.9%)	0 (0%)	0 (0%)	No Obs
Other vegetables	12 (12.0%)	37 (14.0%)	4 (3.0%)	0 (0%)	1 (0.8%)	0 (0%)	94 (25.0%)	28 (7.8%)	15 (14.0%)	1 (20.0%)	3 (4.1%)	No Obs
Other fruits	44 (44.0%)	49 (18.0%)	17 (13.0%)	0 (0%)	0 (0%)	0 (0%)	24 (6.4%)	12 (3.3%)	15 (14.0%)	1 (20.0%)	2 (2.7%)	No Obs

Food groups	Vendor type/income regions											
	Kiosk/duka, *N* = 176^1^			Mom&Pop/cereal shop, *N* = 79^1^			Modern restaurant/fast food outlet/bakery, *N* = 69^1^			Hypermarkets–supermarkets, *N* = 17^1^		
	Low-income	Middle-income	High-income	Low-income	Middle-income	High-income	Low-income	Middle-income	High-income	Low-income	Middle-income	High-income
Grain, roots and tubers	256 (85.0%)	142 (81.0%)	38 (84.0%)	16 (100.0%)	77 (97.0%)	8 (100.0%)	4 (100.0%)	65 (94.0%)	75 (91.0%)	1 (100.0%)	17 (100.0%)	18 (100.0%)
Legumes and pulses	33 (11.0%)	18 (10.0%)	7 (16.0%)	12 (75.0%)	58 (73.0%)	6 (75.0%)	3 (75.0%)	30 (43.0%)	21 (26.0%)	1 (100.0%)	8 (47.0%)	10 (56.0%)
Nuts and seeds	91 (30.0%)	66 (38.0%)	35 (78.0%)	6 (38.0%)	40 (51.0%)	6 (75.0%)	1 (25.0%)	2 (2.9%)	18 (22.0%)	1 (100.0%)	15 (88.0%)	16 (89.0%)
Dairy and dairy products	245 (81.0%)	131 (74.0%)	30 (67.0%)	4 (25.0%)	26 (33.0%)	4 (50.0%)	3 (75.0%)	17 (25.0%)	32 (39.0%)	1 (100.0%)	17 (100.0%)	15 (83.0%)
Meat, poultry and fish	5 (1.7%)	2 (1.1%)	1 (2.2%)	0 (0.0%)	6 (7.6%)	3 (38.0%)	3 (75.0%)	49 (71.0%)	74 (90.0%)	0 (0.0%)	6 (35.0%)	10 (56.0%)
Eggs	207 (69.0%)	128 (73.0%)	28 (62.0%)	5 (31.0%)	28 (35.0%)	6 (75.0%)	2 (50.0%)	29 (42.0%)	44 (54.0%)	1 (100.0%)	17 (100.0%)	15 (83.0%)
Dark green leafy veg	29 (9.6%)	13 (7.4%)	3 (6.7%)	1 (6.3%)	5 (6.3%)	4 (50.0%)	2 (50.0%)	27 (39.0%)	21 (26.0%)	0 (0.0%)	2 (12.0%)	12 (67.0%)
Vitamin A rich fruits and veg	20 (6.6%)	16 (9.1%)	6 (13.0%)	1 (6.3%)	2 (2.5%)	5 (63.0%)	1 (25.0%)	2 (2.9%)	8 (9.8%)	0 (0.0%)	3 (18.0%)	12 (67.0%)
Other vegetables	53 (18.0%)	27 (15.0%)	7 (16.0%)	2 (13.0%)	2 (2.5%)	5 (63.0%)	1 (25.0%)	16 (23.0%)	36 (44.0%)	0 (0.0%)	2 (12.0%)	12 (67.0%)
Other fruits	39 (13.0%)	21 (12.0%)	9 (20.0%)	1 (6.3%)	2 (2.5%)	5 (63.0%)	1 (25.0%)	5 (7.2%)	18 (22.0%)	0 (0.0%)	3 (18.0%)	12 (67.0%)

Food groups	Vendor type/income regions					
	Tabletop/mama mboga/wet market, *N* = 490^1^			Wholesalers, *N* = 11^*1*^		
	Low-income	Middle-income	High-income	Low-income	Middle-income	High-income
Grain, roots, and tubers	129 (46.0%)	221 (45.0%)	35 (51.0%)	10 (77.0%)	3 (27.0%)	3 (100.0%)
Legumes and pulses	29 (10.0%)	54 (11.0%)	9 (13.0%)	3 (23.0%)	2 (18.0%)	1 (33.0%)
Nuts and seeds	11 (3.9%)	3 (0.6%)	11 (16.0%)	5 (38.0%)	0 (0.0%)	2 (67.0%)
Dairy and dairy products	11 (3.9%)	6 (1.2%)	0 (0.0%)	6 (46.0%)	5 (45.0%)	3 (100%)
Meat, poultry and fish	30 (11.0%)	20 (4.1%)	0 (0.0%)	0 (0.0%)	0 (0.0%)	0 (0.0%)
Eggs	28 (9.9%)	39 (8.0%)	19 (28.0%)	4 (31.0%)	4 (36.0%)	1 (33.0%)
Dark green leafy veg	163 (58.0%)	220 (45.0%)	25 (37.0%)	0 (0.0%)	0 (0.0%)	0 (0.0%)
Vitamin A rich fruits and veg	111 (39.0%)	276 (56.0%)	58 (85.0%)	0 (0.0%)	0 (0.0%)	0 (0.0%)
Other vegetables	226 (80.0%)	350 (71.0%)	55 (81.0%)	1 (7.7%)	1 (9.1%)	0 (0.0%)
Other fruits	170 (60.0%)	317 (65.0%)	62 (91.0%)	1 (7.7%)	1 (9.1%)	0 (0.0%)

Furthermore, dark green leafy vegetables (DGLV), which provide essential micronutrients (e.g., Vit. A, folate) were more limited, with only 24% of all vendor types across the three income regions selling them. The highest availability of DGLV is among the farm/home vendors, followed by tabletop vendors/mama mboga/wet market and hypermarkets/supermarkets.

Vitamin A-rich fruits and vegetables were sold predominantly by tabletop vendors/mama mboga/wet markets and hypermarkets/supermarkets. Similarly, other vegetables were sold by 30% of vendor types within the income regions, whereby tabletop vendors (75%) were leading in availability, followed by hypermarkets/supermarkets (39%). Also, for other fruits sold by 25% of food vendors, tabletop vendors (65%) and hypermarkets/supermarkets (42%) have the highest availability.

#### Market Food Diversity differences by Income Regions

The next step involves determining the variation of food group diversity across the income regions of Nairobi using the Market Food Diversity (MFD).

Results in Table 5 show a higher mean MFD observed in the high-income region (3.11), followed by the low-income (2.71) and the middle-income (2.35) respectively. The standard deviations of the low- and middle-income regions are much lower than in high-income. We hypothesized that there are differences in market food diversity across the income regions [37]. We therefore fail to reject the null hypothesis.

**Table 5:** Descriptive statistics of market food diversity

Market food diversity	Mean	Std. Dev	Min	Max
Low-income region	2.71	1.609	1	9
Middle-income region	2.35	1.573	1	10
High-income region	3.11	2.242	1	10

**Fig. 3:**
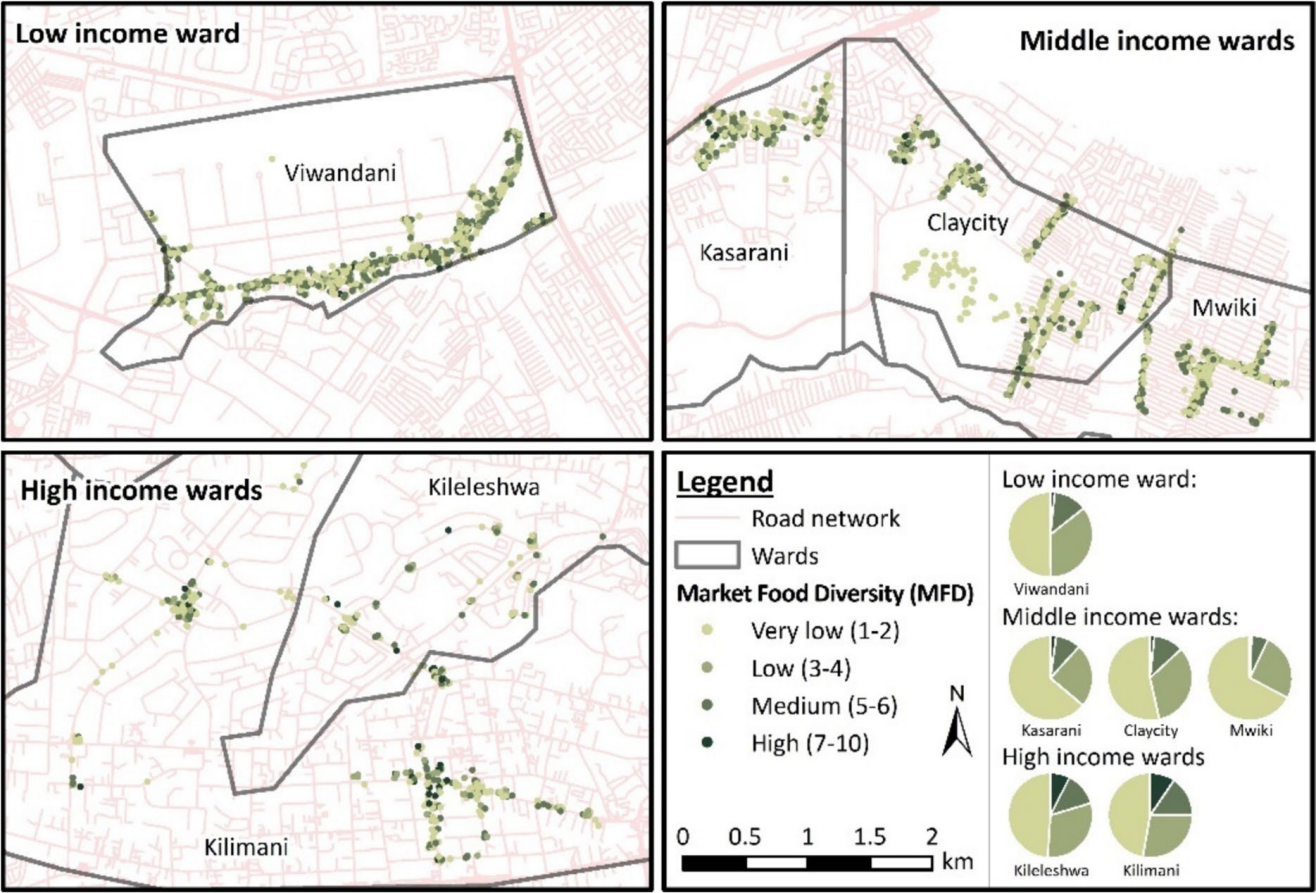
Vendor MFD in high-, middle-, and low-income regions of Nairobi. High-income wards had a higher MFD, followed by middle-income and low-income wards respectively

This is further confirmed in Fig. 3 that shows more food group diversity (darker greens) in the high-income region, followed by low- and middle-income, respectively.

The high-income region overall had most food vendors achieving an MFD of ≥ 5 (22.89%), followed by 14.44% and 10.71% food vendors in low- and middle-income regions, respectively (see Table S3 in Appendix).

**Fig. 4:**
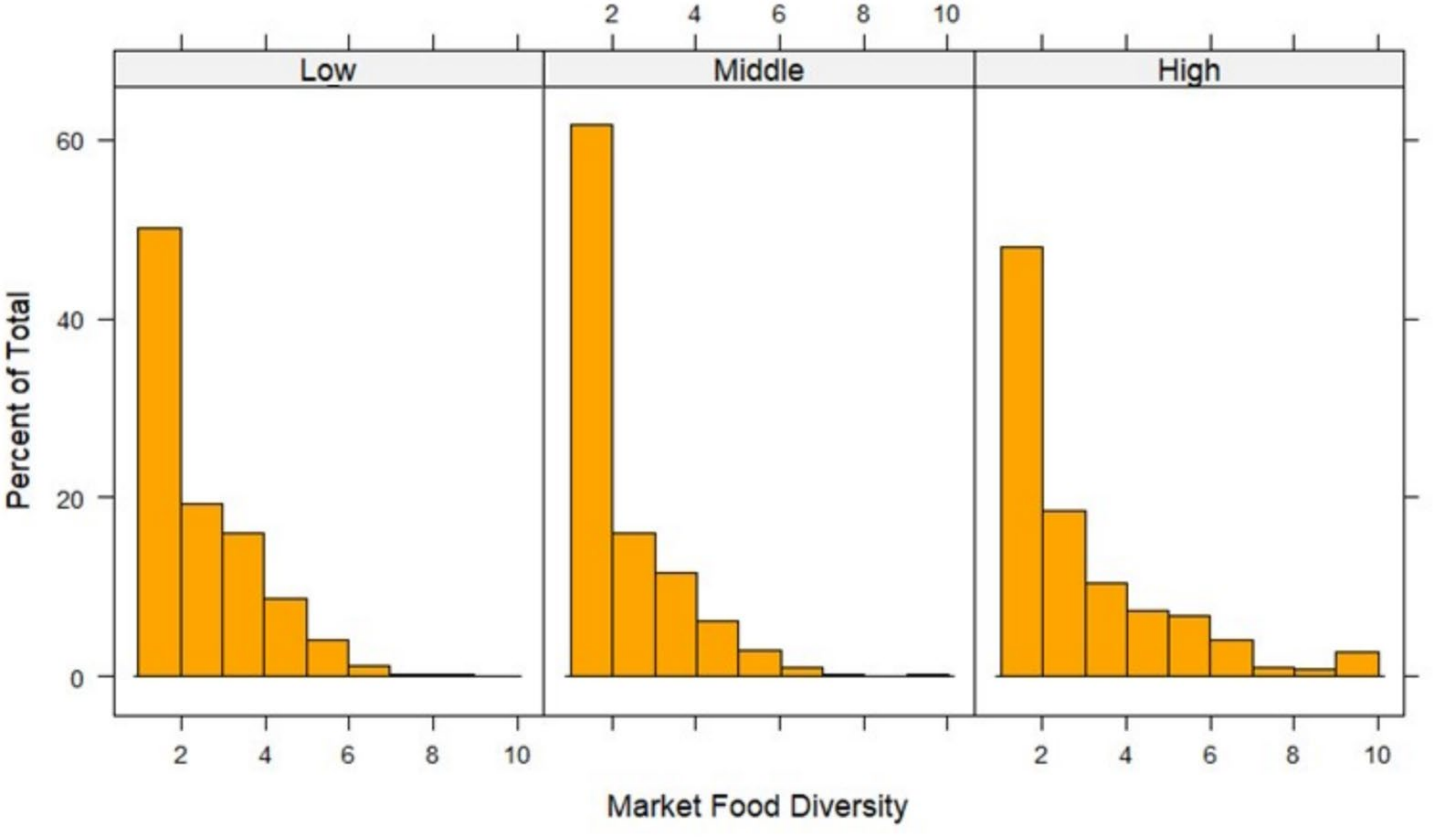
Bar plots showing distribution of MFD of three income regions of Nairobi city. All figures show that MFD distribution is right-skewed

Overall, as shown in Fig. 3, there was a very high proportion of market food diversity below five across the income regions. Very low MFD (1–2) was mostly observed in middle- (61.50%), followed by low- (50.04%) and high-income (48.12%) regions, respectively. The distribution of MFD is also illustrated in the bar plots in Fig. 4, showing right-skewed bar plots of MFD distribution of all income regions.

### High Availability of Unhealthy Foods

We also assessed the differences in prevalence of additional food groups, which are considered by FAO [26] as unhealthy. In this study, these unhealthy foods include condiments and sugary-sweetened beverages, snacks (slaty and sugary such as cakes, sweets), oils and fats, and mixed foods.

**Fig. 5:**
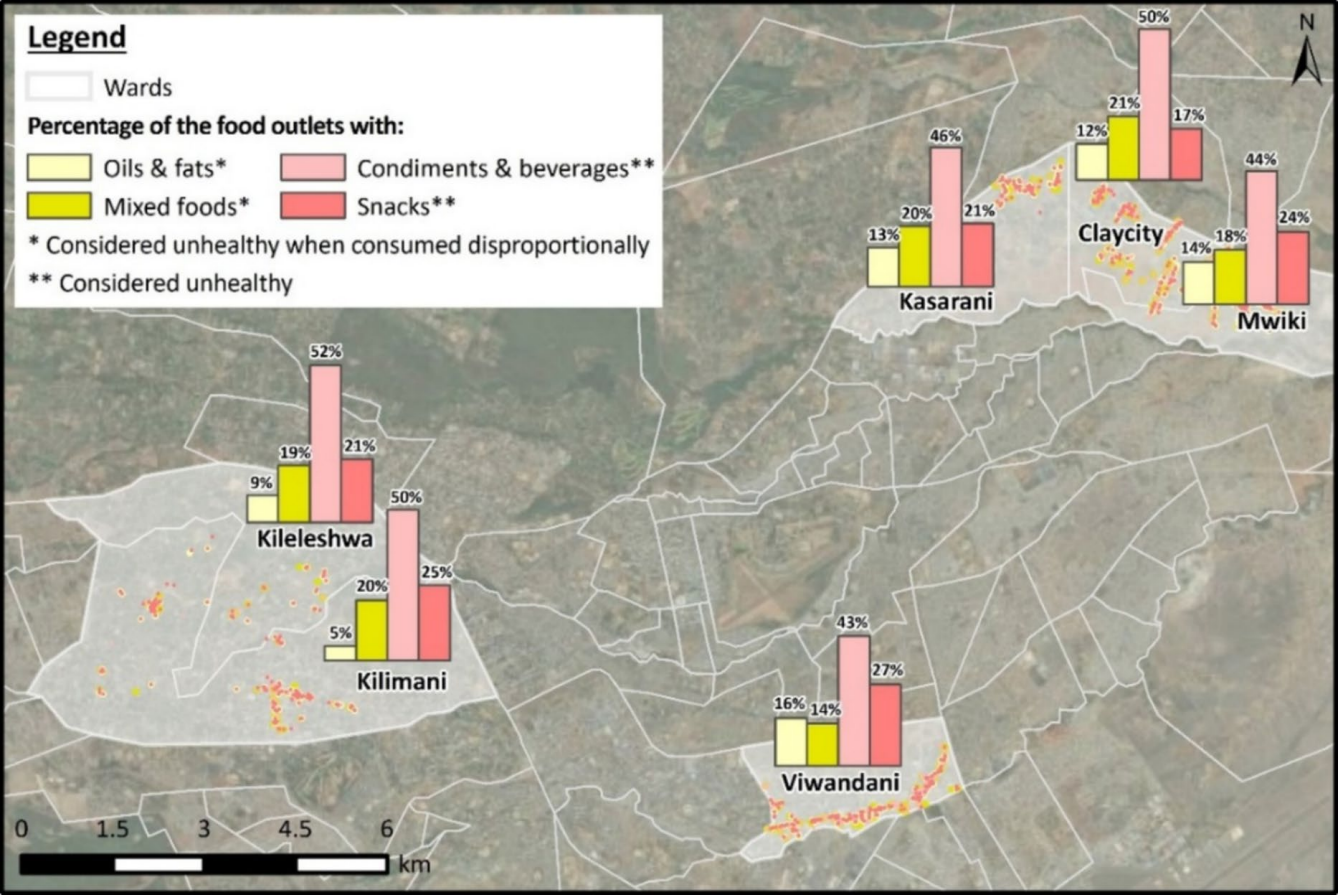
Percentage of food outlets that sell unhealthy food groups such as oils and fats, mixed foods, condiments, and sugary-sweetened beverages and snacks. Mixed foods include fried vegetable/beef samosas, fried bhajia (potato), and fried rice. Condiments include salt-rich food additives; sugary-sweetened beverages include fruit juices, soda, and energy drinks. Snacks include salty crisps, chocolates, and sweets

Figure 5 shows the distribution of unhealthy food across selected the three income regions (within each ward). We focused on oils and fats (light yellow), mixed foods (orange-yellow), condiments and sugary sweetened beverages (light pink), and snacks (dark pink). Each bar shows the percentage of food vendors offering these food groups across the income regions (within each ward). On average, there is a higher percentage of food vendors in the high-income (Kilimani/Kileleshwa) condiments and sugary-sweetened beverages, followed by the middle-income (Kasarani) and low-income (Viwandani) respectively.

### Relationship Between MFD and Income Regions

Table 6 presents the results of the Poisson regression showing the relationship between MFD and income region. Given other factors held constant, the Poisson analysis shows a significant positive relationship between market food diversity and income regions (*p*-value < 0.05). This analysis indicates that income regions are crucial determinants of MFD in urban areas. Our results further show that high-income regions exhibit greater food diversity compared to middle- and low-income regions.

**Table 6:** Poisson regression results explaining factors influencing market food diversity in urban Nairobi

Factors	MFDR^*1*^	95% CI^*1*^	*p*-value
Location of vendor by income region			
High-income	—	—	
Low-income	0.93	0.87, 1.00	0.047
Middle-income	0.82	0.77, 0.87	< 0.001
Vendor ownership/operation			
Joint ownership	—	—	
Single ownership	0.76	0.72, 0.81	< 0.001
Shelf food space			
Below 25%	—	—	
Between 25–50%	1.35	1.10, 1.69	0.006
Between 50–75%	1.88	1.54, 2.33	< 0.001
Between 75–99%	2.08	1.71, 2.56	< 0.001
100% food space	1.63	1.34, 2.00	< 0.001

Significance codes: 0 “***” 0.001 “**” 0.01 “*” 0.05 “.” 0.1 “ “ 1. Null deviance: 3431.4 on 3285 degrees of freedom. Residual deviance: 3090.1 on 3278 degrees of freedom. AIC: 11,876. Shelf food space and vendor ownership significantly influenced market food diversity. Single-ownership vendors had 0.76 lower MFD, while shelf space played a key role in across income regions. Compared to food vendors with less than 25% shelf space, 25–49% space increased MFD by 35%, while 50–74% space raised it by 88%. 75–99% shelf space more than doubled MFD (2.08 times), and 100% space boosted MFD by 1.63 times. This means that larger shelf food space consistently promoted greater market food diversity

^1^*MFDR* market food diversity ratio, *CI* confidence interval

## Discussion

While there are standardized definitions on the global scale contextualizing the definition of the food environment, our study reflects the East African regional context and aims at better local policy framing. Despite our analysis being based on cross-sectional data, causality was not inferred from the associations found. We therefore obtained plausible data to distinguish three food environments in Nairobi, representing most urbanizing cities in LMIC settings.

The first important result is distinguishing between two vendor typologies (formal vs informal). This indicates differences in vendor distribution and food group variation by vendor type. We observed higher diversity among formal vendors, which corresponds to Mwangi et al. [43] who observe that informal vendors barely sold more than one food group. Our results provide a good framework to describe and assess changes within the urban food environment [4], which aims to create policies to improve food security and nutrition. Thus, this study is important for policymakers in designing food policy measures that enable access to healthy and nutritious foods by vendors across different sociodemographic in Nairobi.

Using market food diversity as a metric to measure the food environment is not limited to Nairobi and can be replicated in LMI contexts. For example, studies in South Africa [18] and the USA [30] that observed the predominance of informal and formal vendors, respectively, could use such metrics to measure vendor food group diversity. However, our study acknowledges that diversity is not only within one vendor but could also be at the level of the neighborhood or individuals living in the region.

Similar to our findings, Crush and Frayne [18] reported a mixture of both formal and informal vendors in the middle-income area of South Africa. Moreover, middle-income areas of high-income countries also embrace a mix of formal and informal food vendors that allow a wider variety of food products [54]. The noted higher prevalence of formal food vendors in the high- and middle-income regions could be attributed to superior purchasing power and demand for food services that are formalized [53]. Among other factors, these patterns underscore the notion that income plays a crucial role in shaping the food environment structure with the high-income region supporting a dense concentration of formal food vendors.

The important role of food vendors in driving market diversity and improving diets is reinforced by the vendor (food group) diversity. Across all vendor types, staples illustrate the potential of food vendors to provide key dietary components to urban populations within diverse income regions [58]. Specific food groups such as fish, meat, and poultry are available predominantly among specialized vendors (poultry sellers and butchers) pointing to the expertise of these vendors. This targeting is seen among informal vendors. For example, in Nairobi, meat and poultry are typically sold by vendors who specialize in slaughtering, butchering and preparing these meat products—often operating from permanent stalls (within wet markets) that differentiate them from other food produce sellers [12]. Another target are the tabletop vendors, who play a critical role in the provision of affordable smaller food portions. Tabletop vendors buy in bulk from farmers’ market or wholesalers and resell in manageable smaller portions in low-income regions. A study across five towns (Machakos, Kiambu, Mombasa, Nakuru, and Nairobi) in Kenya found that informal vendors drive market diversity by having a wider range of fresh fruits and vegetables, pulses, eggs, dairy, grains, fish, and meat—driven by the support of local governance structures, e.g., market committees [20].

Some studies have associated higher MFD among higher income residents with access to diverse food options compared to other income regions [67]. In South Africa, favorable urban planning contributed to better infrastructure, which facilitated access to healthier and more diverse food systems among the high-income [5]. Although such patterns are evident, it is important to note that MFD across the food environments does not automatically translate into consumption, but rather individual factors such as food affordability, preferences, and sociocultural practices. Lower MFD in the middle-income region can be attributed to structural issues such as market dynamics that hinder the potential to exhibit greater food diversity compared to the low-income region. In India, low-income food vendors also relied more on local production or farmer markets that were closer in proximity [44].

The predominance of informal vendors highlights their critical role in food security and nutrition across income regions. In low-income urban areas, spatial vendor distribution and economic constraints significantly shape local food environments, determining food availability and access to diverse foods based on proximity. However, while market food diversity is higher in both high- and low-income regions, our results reveal the emergence of food swamps—oversaturated with energy-dense, nutrient-poor foods like snacks and sugary-sweetened beverages (e.g., [17]). For instance, in Kileleshwa, Kilimani, and Clay City wards, over 40% of food vendors sold such unhealthy food options. Even in the high-income region, although healthier foods are prevalent, unhealthy foods remain dominant across socioeconomic study regions. Glanz et al. [28] emphasize how consumer food environments—shaped by product availability, promotion, and pricing—influence individual dietary choices, often leading individuals to opt for unhealthy foods (sugary-sweetened beverages and snacks) due to convenience and affordability.

This trend is consistent across all three income regions—Kileleshwa, Kilimani, Mwiki, Viwandani wards, which mirrors findings by Popkin et al. [50] that urbanization increases access to calorie-dense foods (food swamps) regardless of socioeconomic strata. Compounding this issue, low-income households often rely on small-unit purchases due to limited cash. This makes the *Kadogo* economy—characterized by minimal-quantity buying—essential for survival (e.g., [41]). Consequently, informal vendors adapt by prioritizing immediate, small-scale demands, frequently at the expense of dietary diversity.

Beyond income regions, single ownership by a vendor was associated with a decrease in MFD. Joint ownership brings together different partners with diverse skills, leading to more informed decisions on food product sourcing and packaging, thereby increasing the variety of food groups by a vendor [64].

Anincrease in MFD was associated with more shelf food space provided by the food vendors. Studies show that food vendors with a higher percentage of shelf food space to nutritious food tend to promote healthier food choices among consumers [45].

## Conclusion

This quantitative study shows new evidence in assessing market food diversity across different demographics in an LMIC setting in Kenya. Comparison across three income regions provides a novel contribution to the emerging body of literature on food environment studies. Unlike other studies conducted in many LMICs that focus on low-income regions, this research focusses on low-, middle-, and high-income regions. The study demonstrates the significant role of the built food environment in showing differences in vendor characteristics and distribution, food accessibility, and vendor food group diversity across income regions.

Our results show that food vendors across the income regions sold unhealthy foods, which highlights a shared exposure across the diverse neighborhoods. This provides an opportunity for a deeper understanding of the changes in the built FE, and how these changes would impact urban policymaking on support for informal food vendors and regulations on food safety. The differences in market diversity across the neighborhoods reveal the urgency to design interventions, e.g., nutrition-focused vendor training targeted at promoting access to diverse diets for populations.

While diversity has been measured in this study, the dimensions of food prices and food safety and waste are equally important. Future empirical studies should consider comparing these other external food dimensions to provide a more deeper understanding of the food environment in LMIC. The study focused on the built FE, but for a more holistic understanding of the FE, future research should examine the interaction between the built FE and consumer behavior, particularly how purchasing power and individual preferences influence food choices and access.

This study used market food diversity to measure vendor diversity. However, future studies should consider more metrics (e.g., cost of healthy diets) to measure other dimensions of the food environment to gain more regional contextualized knowledge and understanding in the urban LMIC context.

## Supplementary Information

Below is the link to the electronic supplementary material.

Supplementary file1 (DOCX 55 KB)

## Data Availability

The data will be available upon request from the corresponding author.

## Footnotes

1 A woman who sells fruits and vegetables, including other food items on open-air markets, along the roadside stalks or small shops.
