# Supplementary material for: Assessing Market Food Diversity of Three Food Environments of Nairobi, Kenya, Using Spatial and Descriptive Analyses
Source: J Urban Health. 2025 Sep 8;102(4):742–59. doi: 10.1007/s11524-025-00999-4 (PMC12484525; doi:10.1007/s11524-025-00999-4)
Supplement: Supplementary file 1 — Supplementary file1 (DOCX 55 KB) [file 11524_2025_999_MOESM1_ESM.docx]

## Appendix

Table S 1. Aggregated subdivision of food groups for MFD index

| **Assigning questionnaire rows to the 10 MDD-W food groups** | | |
| --- | --- | --- |
|  | **Questionnaire rows** | **Food groups** |
| A.  B. | Grains and cereals  Roots and tubers | 1. Grains, white roots and tubers, and plantains |
| C. | Legumes and pulses | 1. Legumes and pulses |
| D. | Nuts and seeds | 1. Nuts and seeds |
| E. | Dairy products | 1. Dairy products |
| F.  G.  H.  I. | Flesh meat  Organ meat  Poultry  Fish | 1. Meat, poultry and fish |
| J. | Eggs | 1. Eggs |
| K. | Dark green leafy vegetables | 1. Dark green leafy vegetables |
| L.  M. | Vitamin A-rich fruits and vegetables  Vitamin A-rich fruits | 1. Vitamin A-rich fruits and vegetables |
| N. | Other vegetables | 1. Other vegetables |
| O. | Other fruits | 1. Other fruits |

**Source:** (FAO, 2021)

Table S 2. GLM in estimating the robustness of the Poisson model

| **Factors** | **exp(Beta)** | **95% CI***^1^* | **p-value** |
| --- | --- | --- | --- |
| **Location of vendor by income region** |  |  |  |
| High | — | — |  |
| Low | 0.80 | 0.66, 0.97 | **0.020** |
| Middle | 0.57 | 0.48, 0.68 | **<0.001** |
| **Vendor ownership/ operation** |  |  |  |
| joint ownership | — | — |  |
| single ownership | 0.44 | 0.37, 0.53 | **<0.001** |
| **Shelf food space** |  |  |  |
| Below 25% | — | — |  |
| Between 25% - 50% | 1.72 | 1.10, 2.68 | **0.018** |
| Between 50% - 75% | 3.80 | 2.48, 5.82 | **<0.001** |
| Between 75% - 99% | 5.16 | 3.40, 7.83 | **<0.001** |
| 100% food space | 2.59 | 1.73, 3.89 | **<0.001** |
| *^1^*CI = Confidence Interval  Signif. codes: 0 ‘***’ 0.001 ‘**’ 0.01 ‘*’ 0.05 ‘.’ 0.1 ‘ ’ 1  Null deviance: 9658.1 on 3285 degrees of freedom  Residual deviance: 8747.1 on 3278 degrees of freedom  AIC: 12560 | | | |

Table S 3. Market Food Diversity derived from the MDD-W index

| **Food Group Scaling** | **Low-income region (%)** | **Middle-income region (%)** | **High-income region (%)** |
| --- | --- | --- | --- |
| 1 | 32.33 | 43.62 | 32.42 |
| 2 | 17.71 | 17.87 | 15.68 |
| 3 | 19.38 | 16.06 | 18.43 |
| 4 | 16.12 | 11.73 | 10.59 |
| 5 | 8.72 | 6.26 | 7.42 |
| 6 | 4.05 | 2.89 | 6.78 |
| 7 | 1.23 | 1.08 | 4.03 |
| 8 | 0.26 | 0.24 | 1.06 |
| 9 | 0.18 | 0.06 | 0.85 |
| 10 | 0 | 0.18 | 2.75 |
| Total (%) | 100 | 100 | 100 |

**Food Environment Mapping Tool: Food Vendor Inventory**

Date of the survey completion (dd/mm): ___/____

Name / surname of the enumerator: ___________________/_______________

County: _____________________________

Sub-county: __________________________

Ward/ village: ______________________________

Neighborhood or village name: ___________________

RECORD THE **LOCATION OF THE OUTLET** USING GPS INFORMATION

Geographic position of the outlet: ________ dg __________ mn latitude (East) and __________ dg ______ mn longitude (North)

**GENDER** OF THE VENDOR

0= Female

1= Male

2= Females

3= Males

4= Both male and female

How much of the vendor space is food?

1= Less than 25%

2= 25% - 50%

3= 50% - 75%

4= Above 75%

5= 100%

| **CATEGORIZATION**  VISUALY DETERMINE THE TYPE OF OUTLET - if you are uncertain and/or hesitate between two categories, tick both.   \| Vendor category \| Select \| Description \| \| --- \| --- \| --- \| \| 1. Ambulant vendor/street hawkers/mobile vendors \|  \|  \| \| 2. kiosk/retail shop \|  \|  \| \| 3. Street / sidewalk catering/ vendor \|  \|  \| \| 4. Home vendor \|  \|  \| \| 5. Wet market/ open air market \|  \|  \| \| 6. Butcher \|  \|  \| \| 7. Small or medium size “modern” restaurant/ catering \|  \|  \| \| 8. Poultry \|  \|  \| \| 9. Supermarket \|  \|  \| \| 10. Fast food outlets \|  \|  \| \| 11. Direct sale from the farm \|  \|  \| \| 12. Wholesalers \|  \|  \|   **VISUAL INVENTORY OF THE OUTLET**  VISUALY DETERMINE THE TYPE OF ITEMS THAT ARE ON SELL IN THE OUTLET | | |
| --- | --- | --- | --- | --- | --- | --- | --- | --- | --- | --- | --- | --- | --- | --- | --- | --- | --- | --- | --- | --- | --- | --- | --- | --- | --- | --- | --- | --- | --- | --- | --- | --- | --- | --- | --- | --- | --- | --- | --- | --- | --- |
| **Food Groups** | **Availability**  **yes/no** | **Color for every item related to fruits and vegetables and roots only.**  1= Dark leafy green  2= Green (other)  3= Red  4= Yellow, Orange  5= Purple, Blue  6= White, Brown |
| **Grains** | | |
| Bread (white) |  |  |
| Bread (brown) |  |  |
| Breakfast cereal (wheetabix, oatmeal, etc.) |  |  |
| Chapati (white) |  |  |
| Chapati (brown) |  |  |
| Maize (green) |  |  |
| Maize (roasted or boiled) |  |  |
| Maize (dry) |  |  |
| Maize flour (shifted) |  |  |
| Maize flour (whole grain) |  |  |
| Mandazi |  |  |
| Millet (dry grain) |  |  |
| Millet flour |  |  |
| Noodles |  |  |
| Porridge (sorghum) |  |  |
| Porridge (white flour) |  |  |
| Rice (raw) |  |  |
| Rice (boiled/fried) |  |  |
| Scones |  |  |
| Sorghum (dry grains) |  |  |
| Sorghum flour |  |  |
| Spaghetti |  |  |
| Ugali (shifted maize) |  |  |
| Ugali (whole grain) |  |  |
| Ugali (millet flour) |  |  |
| Wheat flour (shifted) |  |  |
| Wheat flour (whole grain) |  |  |
| Other grains (specify) |  |  |
|  |  |  |
| **White roots and tubers, and plantains** | |  |
| Arrow roots |  |  |
| Arrow roots (crisps) |  |  |
| Green banana/plantain |  |  |
| Green banana/plantain crisps |  |  |
| Cassava (raw) |  |  |
| Cassava (boiled/ roasted) |  |  |
| Cassava crisp |  |  |
| Green banana/Plantain |  |  |
| Irish potato |  |  |
| Irish potato chips |  |  |
| Irish potato crisps |  |  |
| Sweet potato (raw) |  |  |
| Sweet potato (Boiled, roasted) |  |  |
| Yam |  |  |
| Other roots and tubers (specify) |  |  |
|  |  |  |
| **Pulses** |  |  |
| Beans (raw) |  |  |
| Beans (boiled/roasted) |  |  |
| Chickpeas |  |  |
| Cowpea |  |  |
| Dolichos (njahe) (raw) |  |  |
| Dolichos (njahe) (Boiled) |  |  |
| Lentils |  |  |
| Mung beans |  |  |
| Pigeon peas |  |  |
| Peas (Minji) (Raw) |  |  |
| Peas (Minji) (cooked) |  |  |
| Soybeans |  |  |
| Other pulses (specify) |  |  |
|  |  |  |
| **Nuts and seeds** |  |  |
| Almonds (raw) |  |  |
| Almonds (roasted) |  |  |
| Cashews (raw) |  |  |
| Cashews (roasted) |  |  |
| Coconut flesh |  |  |
| Groundnut/peanut (raw) |  |  |
| Groundnut/peanut (roasted) |  |  |
| Macadamia nuts (raw) |  |  |
| Macadamia nuts (roasted) |  |  |
| Peanut butter |  |  |
| Pumpkin seed (raw) |  |  |
| Pumpkin seed (roasted) |  |  |
| Sesame seeds (raw) |  |  |
| Sesame seeds (roasted) |  |  |
| Sunflower seed |  |  |
| Other nuts and seeds (specify) |  |  |
|  |  |  |
| **Dairy and Dairy products** |  |  |
| Cheese |  |  |
| Cream |  |  |
| Fermented milk (mala, Kefir) |  |  |
| Ghee |  |  |
| Milk |  |  |
| Yoghurt |  |  |
| Other dairy products |  |  |
|  |  |  |
| **Meat, poultry and fish** |  |  |
| Beef - cow meat (raw) |  |  |
| Beef - cow meat (boiled/roasted/fried) |  |  |
| Burger |  |  |
| Chicken (raw) |  |  |
| Chicken (boiled/roasted/fried) |  |  |
| Duck (raw) |  |  |
| Duck (boiled/roasted/fried) |  |  |
| Fish – whole or fillet (raw) |  |  |
| Fish – whole or fillet (boiled/roasted/fried) |  |  |
| Goat meat (raw) |  |  |
| Goat meat (boiled/roasted/fried) |  |  |
| Insects (e.g., Locust, grasshoppers, termites, giant water bug) |  |  |
| Lamb - sheep meat (raw) |  |  |
| Lamb – sheep meat (boiled/roasted/fried) |  |  |
| Matumbo (intestines) |  |  |
| Mutura |  |  |
| Omena (raw) |  |  |
| Omena (boiled/fried) |  |  |
| Organ meat (raw) |  |  |
| Organ meat (boiled/roasted/fried) |  |  |
| Pigeon (raw) |  |  |
| Pigeon (boiled/roasted/fried) |  |  |
| Pork - pig meat (raw) |  |  |
| Pork - pig meat (boiled/roasted/fried) |  |  |
| Sausage (raw) |  |  |
| Sausage (boiled/roasted/fried) |  |  |
| Turkey (raw) |  |  |
| Turkey (boiled/roasted/fried |  |  |
| Other meat, poultry and fish products |  |  |
|  |  |  |
| **Eggs** |  |  |
| Eggs (raw) |  |  |
| Eggs (boiled/fried) |  |  |
| Other egg products (specify) |  |  |
|  |  |  |
| **Dark green leafy vegetables** |  |  |
| Spinach |  |  |
| Kales (sukuma wiki) |  |  |
| Mrenda (jute mallow) |  |  |
| Kunde |  |  |
| Kanzera (collard greens) |  |  |
| Managu (African night shade) |  |  |
| Sagaa (spider plant) |  |  |
| Pumpkin leaves |  |  |
| Cowpea leaves |  |  |
| Terere (amaranthus) |  |  |
| Mitoo (slender leaves) |  |  |
| Other dark green vegetables (specify) |  |  |
|  |  |  |
| **Other vitamin A-rich fruits and vegetables** |  |  |
| Butternut |  |  |
| Carrots |  |  |
| Deep yellow- or orange-fleshed squash |  |  |
| Orange fleshed sweet potatoes |  |  |
| Pumpkin |  |  |
| Red sweet capsicum |  |  |
| Ripe mango |  |  |
| Ripe papaya |  |  |
| Other Vitamin A rich fruits and vegetables (specify) |  |  |
|  |  |  |
| **Other vegetables** |  |  |
| Cabbage |  |  |
| Cucumber |  |  |
| Eggplant |  |  |
| Tomato |  |  |
| Green sweet capsicum |  |  |
| Mushrooms |  |  |
| Onions (purple/ white/ grass onions) |  |  |
| Green beans |  |  |
| French beans |  |  |
| Okra |  |  |
| Other vegetables (specify) |  |  |
|  |  |  |
| **Other fruits** |  |  |
| Avocado |  |  |
| Ripe banana |  |  |
| Oranges |  |  |
| Tamarind |  |  |
| Jackfruit |  |  |
| Watermelon |  |  |
| Pineapple |  |  |
| Pears |  |  |
| Lemon |  |  |
| Guava |  |  |
| Kiwi |  |  |
| Other fruits (specify) |  |  |
| **Oils and fats** |  |  |
| Cooking oil (elianto, Fresh fri, sunshine, rina, Bahati, Nyota) |  |  |
| Margarine |  |  |
| Butter |  |  |
| Lard |  |  |
|  |  |  |
| **Mixed dishes** |  |  |
| Githeri (Maize+Beans) |  |  |
| Mokimo (Potato+Maize+Vegetable) |  |  |
| Vegetable Samosa (Vegetable + Wheat) |  |  |
| Beef Samosa (Meat+ Wheat) |  |  |
| Vegetable fried rice/vegetable |  |  |
| Beef fried rice/pilau |  |  |
| Meat (mixed with potato+carrots) |  |  |
| **Condiments, seasonings and beverages** |  |  |
| Fresh or dried herbs |  |  |
| Royco (beef and chicken) |  |  |
| Spices (pilau masala, tea masala, coriander powder, chillies, royco cubes) |  |  |
| Ketchup, mustard, pepper sauce |  |  |
| Soy sauce |  |  |
| Tomato paste |  |  |
| Sweetened beverages (coke, pepsi) |  |  |
| Energy drinks |  |  |
| Alcoholic beverages (beer, wine) |  |  |
| Other condiments, seasoning and beverages (specify) |  |  |
|  |  |  |
| **Snacks (savoury and fried snacks, sweets)** |  |  |
| Biscuits |  |  |
| Canned fruits |  |  |
| Cakes and pastries |  |  |
| Candies (fruit gummy candies) |  |  |
| Cookies |  |  |
| Chocolates |  |  |
| Ice-cream |  |  |
| Pie |  |  |
| Sweets |  |  |
| Other snacks (specify) |  |  |

TAKE A **PICTURE OF THE OUTLET**

Picture taken : 1= Yes / 0= No
